# Research Advances in Diagnostic Methods for Prevalent Neurological Diseases

**DOI:** 10.3390/bios16070368

**Published:** 2026-07-06

**Authors:** Mengli Lv, Xiaojie Sun, Xinpeng Wang

**Affiliations:** 1 School of Pharmacy, Qingdao University, Qingdao 266071, China; 2 Qingdao Central Hospital, School of Rehabilitation Sciences and Engineering, University of Health and Rehabilitation Sciences, Qingdao 266113, China; 3 State Key Laboratory of Intelligent Manufacturing Equipment and Technology, Huazhong University of Science and Technology, Wuhan 430074, China

**Keywords:** neurological disease, detection methods, biomarkers, diagnosis and sensing, multimodal integration

## Abstract

Global population aging has emerged as a major driver of the growing burden of neurological diseases, highlighting the urgent demand for advances in early diagnosis, prevention, and rehabilitation. These conditions are typically characterized by insidious onset and irreversible progression, yet their clinical management remains critically compromised by substantial diagnostic delays, representing an intractable bottleneck for existing detection technologies. Therefore, the development of precise, early-stage detection technologies is crucial for expanding the therapeutic window and improving long-term clinical outcomes, addressing a critical unmet clinical need. Herein, we review and compare precision detection strategies for neurological diseases, focusing on the types and mechanisms of mainstream biosensing platforms. Based on the classification of detection substrates and signal transduction mechanisms, four major bio-detection branches are analyzed, including liquid, exosomal, imaging, and digital biomarker detection, with representative studies demonstrating detection limits reaching femtomolar concentrations, clinical diagnostic sensitivities exceeding 90%, and classification accuracies comparable to or surpassing conventional imaging modalities. The inherent advantages and limitations of each biosensing technology are also comprehensively discussed. This review underscores that future research on neurological biomarker sensing is trending toward multimodal integration, which enables the construction of more robust early warning and prognostic assessment systems. This work aims to provide valuable theoretical insights for clinical translation of relevant sensing technologies and integrated diagnostic and treatment strategies, thereby facilitating the progress of early intervention and rehabilitation for common neurological diseases.

## 1. Introduction

Brain health has emerged as a pressing global priority, driven by the escalating burden of neurological diseases that now constitute a major public health crisis. The Global Burden of Disease Study 2021 illuminates that neurological conditions impact approximately 3.4 billion individuals worldwide, representing 43.1% of the global population [1], which demands urgent innovation in bio-detection technologies for early detection and intervention. Neurological diseases can be scientifically classified according to pathological characteristics and pathogenesis, thereby facilitating the identification of shared pathogenic features to optimize targeted diagnostic strategies.

Firstly, neurotrauma and stroke-related diseases cover spinal cord injury, traumatic brain injury, concussion, and ischemic stroke. These diseases share similar pathological changes, therapeutic principles, and diagnostic demands, affecting an extremely large population worldwide [2]. Sudden cerebrovascular occlusion in stroke causes irreversible infarction and necrosis within minutes, accompanied by severe tissue destruction, extensive neuroinflammation, and cerebral edema [3]. Such pathological differences provide important guidance for the development of cerebrospinal fluid (CSF) and blood-based diagnostic testing. Secondly, typical neurodegenerative diseases including Alzheimer’s Disease (AD), Parkinson’s Disease (PD), Frontotemporal Dementia (FTD), and Amyotrophic Lateral Sclerosis (ALS) fall into an independent category [4]. These diseases share core pathogenic commonalities, such as chronic neuroinflammation, ultra-long disease course, and impaired reactive astrogliosis [5].

Thirdly, multiple sclerosis, epilepsy, bipolar disease, and major depressive disease belong to neuroinflammation-associated neurological and psychiatric diseases, whose core shared pathogenesis is persistent neuroinflammatory response driving disease onset and progression [6]. Fourthly, central nervous system tumors consist of intrinsic tumors (glioblastoma, neuroblastoma, oligodendroglioma) and metastatic tumors, with unique diagnostic and clinical characteristics [7]. Given the substantial burden of these neurological diseases, precise bio-detection has become a critical prerequisite for precision therapeutics.

**Figure 1:**
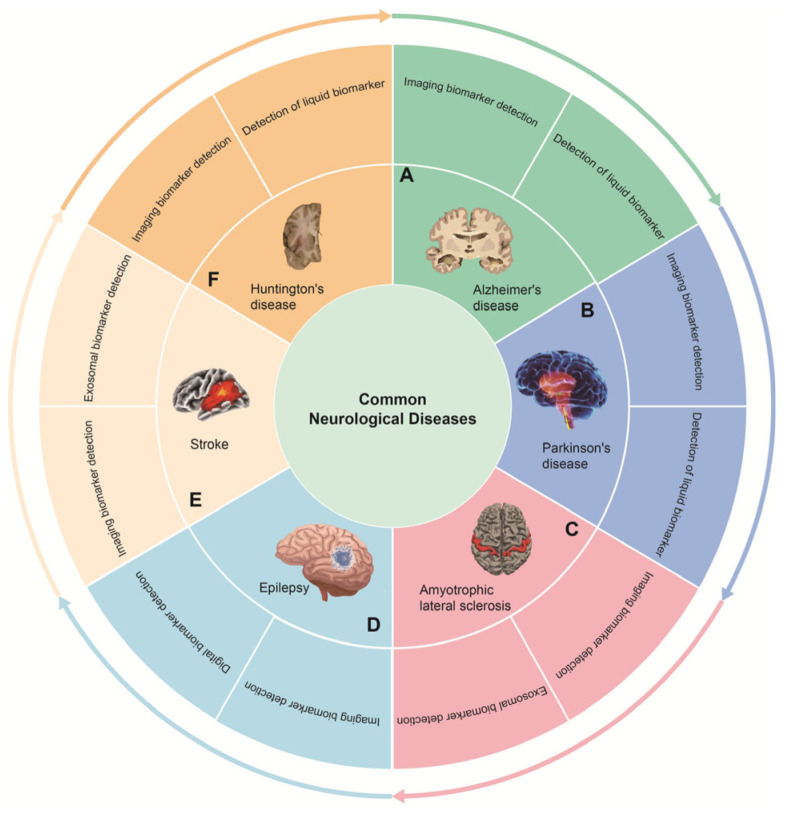
Classification of common neurological diseases and their principal detection methods. (**A**) Alzheimer’s disease. Adapted from Fymat [8], CC BY 4.0. (**B**) Parkinson’s disease. Adapted from Fymat [8], CC BY 4.0. (**C**) Amyotrophic lateral sclerosis. Adapted from Ilieva et al. [9], CC BY-NC.4.0. (**D**) Epilepsy. Adapted from Li et al. [10], CC BY 4.0. (**E**) Stroke. Adapted from Jiang et al. [11], CC BY 4.0. (**F**) Huntington’s disease. Adapted from Bakels et al. [12], CC BY-NC-ND 4.0.

In recent years, based on the classification of detection substrates and signal transduction mechanisms, bio-detection research has been categorized into four main directions, including liquid, exosomal, imaging, and digital biomarker detection. Each category possesses distinct advantages and limitations in signal capture and analysis. Strategic selection of the appropriate bio-detection method matched to specific clinical scenarios enables pre-symptomatic intervention, therapeutic guidance, and reduced disease progression and incidence. This narrative review provides a comprehensive overview of the classifications and pathological mechanisms of common neurological diseases, analyzes the advantages and disadvantages of different bio-detection methodologies, and offers theoretical insights for the clinical translation of pertinent technologies and diagnostic–therapeutic strategies, as illustrated in Figure 1. By synthesizing representative studies selected for their topical relevance and methodological significance, this work aims to highlight emerging trends and critical challenges in neurological bio-detection rather than to provide an exhaustive evidence synthesis. Moreover, future perspectives are proposed to offer new insights for innovative research and to promote advances in human health and rehabilitation medicine.

## 2. Types and Mechanisms of Several Common Neurological Diseases

Neurological diseases are defined by the progressive dysfunction and loss of neurons [13]. Hallmark features include protein misfolding, aggregation, and accumulation, leading to cellular dysfunction, synaptic connectivity loss, and cerebral injury [14]. The principal neurological diseases, along with their pathogenic mechanisms, are summarized below based on their distinct pathological and clinical manifestations.

### 2.1. Alzheimer’s Disease

**Figure 2:**
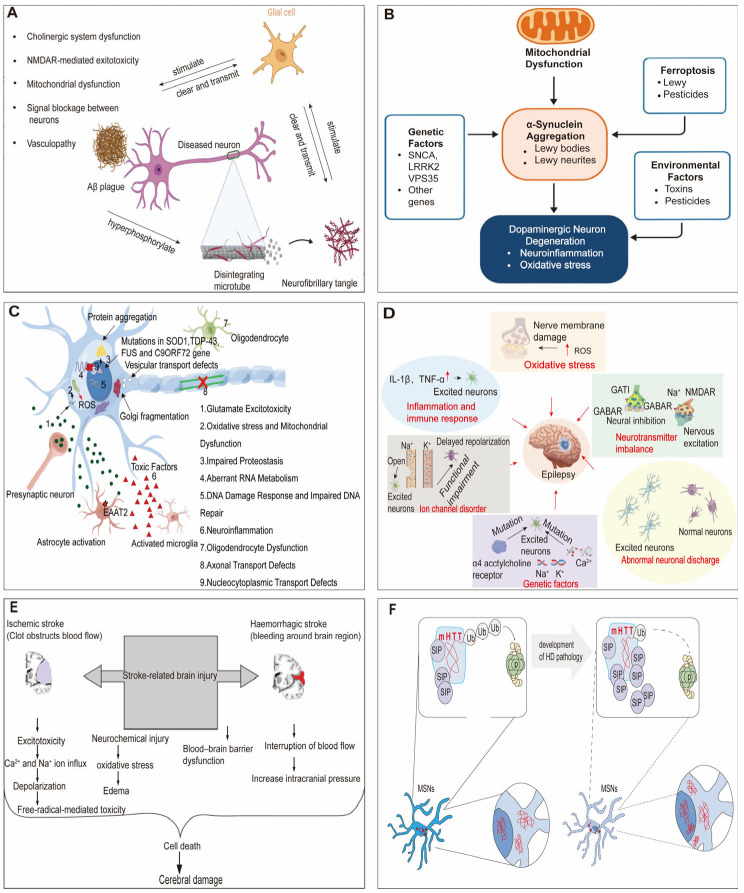
Mechanistic overview of several common neurological diseases. (**A**). In Alzheimer’s Disease, Aβ promotes tau pathology and cholinergic dysfunction, and together with tau activates glial cells, jointly driving pathological neuronal changes. Reproduced from Zhang et al. [22], CC BY 4.0. (**B**). Schematic of dopaminergic neuron degeneration in Parkinson’s Disease. Reproduced from Chaudhary et al. [30], CC BY 4.0. (**C**). Schematic of pathological mechanisms in amyotrophic lateral sclerosis (ALS). Adapted from Hu et al. [56], CC BY-NC-SA.4.0 (**D**). Illustration of how epileptiform discharges amplify inflammation via chemokine storms, blood-brain-barrier disruption, and excitation-inhibition imbalance. Adapted from Li et al. [10], CC BY 4.0. (**E**). Schematic of stroke-related brain-injury mechanisms. Adapted from Kuriakose and Xiao [46], CC BY 4.0. (**F**). Schematic of mutant huntingtin protein aggregation and impaired clearance in Huntington’s disease. Adapted from Latoszek et al. [54], CC BY 4.0.

AD is defined by degenerative alterations in brain cells and represents the leading cause of dementia, characterized by progressive cognitive decline and impaired activities of daily living [15]. The prevalence of AD among individuals aged 60 and above in China is approximately 5.4%, reflecting the challenges posed by population aging [16]. The primary pathological mechanism of AD centers on the interplay between amyloid-beta (Aβ) and tau proteins. Abnormal Aβ accumulation drives sequential tau phosphorylation at distinct epitopes, each exhibiting stage-specific diagnostic utility: p-tau231 deviates earliest during the pre-amyloid phase [17,18], p-tau181 tracks early amyloid burden [19], and p-tau217 achieves optimal accuracy across all disease stages, with the strongest association with advanced tau pathology (Braak stages V–VI) and MCI-to-dementia conversion [20,21]. In terms of pathological progression, this Aβ-driven tau phosphorylation cascade culminates in hyperphosphorylated tau detachment, aggregation, and subsequent neurofibrillary tangle (NFT) formation. Beyond tau pathology, Aβ also contributes to cholinergic system dysfunction and other neurotoxic processes, collectively producing pathologically modified neurons (Figure 2A) [22]. Within this pathological framework, dysregulation of regulatory proteins further modulates tau aggregation. For instance, Zhang et al. [23] demonstrated that TRIM11 functions as an endogenous tau suppressor, directly binding to and degrading hyperphosphorylated tau to inhibit its aggregation. Marked downregulation of TRIM11 in AD brain tissues thereby facilitates NFT formation, highlighting a critical regulatory node in disease progression.

### 2.2. Parkinson’s Disease

PD is a prototypical movement disease, clinically characterized by resting tremor, muscular rigidity, and bradykinesia, with intracellular aggregation of α-synuclein serving as its pathological hallmark [24]. It is the second most prevalent neurodegenerative disease, with an increasing global incidence. The current worldwide patient population exceeds 6 million, and projections suggest that this number will reach 12–17 million by 2040. By 2050, the prevalence is expected to rise further to 25.2 million, reflecting a 112% increase from 2021, which will significantly escalate the disease burden [25,26,27]. In 1817, James Parkinson, a notable British physician, coined the term “PD”, initially referred to as “paralysis agitans” or “shaking palsy” [28,29]. This condition was later characterized as a debilitating neurodegenerative disease of the motor system, arising from multifactorial etiopathogenesis. Genetic mutations, such as those in SNCA, LRRK2, and VPS35, promote α-synuclein aggregation and the development of characteristic Lewy bodies and Lewy neurites. Mitochondrial dysfunction further intensifies protein misfolding and injury induced by oxidative stress. Exogenous factors, including environmental toxins and pesticides, trigger ferroptosis and hasten synuclein aggregation, thereby exacerbating pathological progression. These interrelated pathogenic pathways ultimately converge to trigger neuroinflammation and oxidative stress responses, leading to progressive neuronal degeneration (Figure 2B) [30]. Zou et al. [31] reported that loss of ATP13A2 function disrupts mitochondrial homeostasis and activates the NLRP3 inflammasome, thereby exacerbating neuroinflammation and dopaminergic neuronal injury, offering novel insights into the pathogenesis of PD.

### 2.3. Amyotrophic Lateral Sclerosis

ALS is a progressive neurodegenerative disease characterized by the degeneration of motor neurons in the primary motor cortex, corticospinal tracts, brainstem, and spinal cord [32]. This selective motor neuron loss leads to progressive muscle weakness, dysarthria, expressive language difficulties, and varying degrees of linguistic impairment [33,34]. The pathogenesis of ALS involves multiple converging mechanisms, including glutamate-induced excitotoxicity, oxidative stress, mitochondrial dysfunction, proteostasis imbalance, RNA metabolism dysregulation, nucleocytoplasmic transport defects, impaired DNA damage repair, neuroinflammation, oligodendrocyte dysfunction, and disrupted axonal transport (Figure 2C) [35]. From a diagnostic perspective, given the genetic heterogeneity of ALS, sporadic and familial subtypes require distinct biomarker strategies. SOD1-mutant ALS features mutant protein aggregation, detectable by CSF SOD1 protein assays [36] or seed amplification assays [37]. FUS-mutant ALS exhibits cytoplasmic mislocalization, necessitating FUS oligomerization markers [38]. C9orf72-mutant ALS is characterized by dipeptide repeat accumulation, measurable through poly-GP/GA proteins in CSF [39]. No universal ALS biomarker currently exists; thus, genetic stratification precedes mutation-specific confirmation. These interconnected pathological mechanisms collectively mediate progressive motor neuron degeneration. For instance, Zhao et al. [40] demonstrated that TDP-43 can activate microglial CD14 receptors, NF-κB, and the NLRP3 inflammasome, initiating pro-inflammatory signaling pathways that exacerbate motor neuronal injury.

### 2.4. Epilepsy

Epileptic seizures result from abnormal, excessive, or synchronized neuronal discharges in the brain, representing a cerebral disease characterized by paroxysmal manifestations [41]. It is among the most prevalent serious neurological diseases, impacting individuals of all age groups worldwide. As of 2021, the global population of epilepsy patients reached 51.7 million [42]. The pathophysiological mechanisms involved include ion channel dysfunction, neuronal injury, activation of inflammatory responses, remodeling of synaptic plasticity, aberrant proliferation of glial cells, and mossy fiber sprouting [43]. Schematic illustrations of multi-level interactions demonstrate that the core pathogenesis of epilepsy consists of three principal components: abnormal neuronal discharges, excitation-inhibition imbalance in neural circuits, and ion channel dysfunction. These elements are closely linked and synergistically are influenced by genetic susceptibility, neuroinflammation, and oxidative stress-induced injury, collectively propelling disease progression (Figure 2D) [10]. Song et al. [44] experimentally validated that mitochondrial damage induces mitochondrial DNA release, which activates the cGAS-STING pathway, driving astrocytic production of D-serine and enhancing neuronal excitability, thereby exacerbating seizure severity; inhibition of this pathway attenuates epileptic activity.

### 2.5. Stroke

Stroke, commonly known as a “brain attack”, is divided into two main types: ischemic stroke and hemorrhagic stroke. It is characterized by acute focal neurological deficits caused by vascular damage in the central nervous system, either through infarction or hemorrhage [45]. According to statistics from 2019, stroke was the primary cause of disability and the second leading cause of mortality among cerebrovascular diseases worldwide. The primary pathogenic cascade in stroke is characterized by the collapse of the “neurovascular unit”, which is initiated by the interruption of cerebral blood flow. Hematoma compression or thromboembolism leads to an immediate reduction in regional cerebral perfusion, which subsequently triggers excitotoxicity, ionic overload, microglial activation, and free radical cascades. These processes rapidly disrupt the blood–brain barrier and induce vasogenic edema, while progressively increasing oxidative stress and the release of inflammatory cytokines. Ultimately, these events culminate in irreversible neuronal death, resulting in either ischemic or hemorrhagic injury (Figure 2E) [46]. In the aforementioned ionic disturbances and cellular injury processes, the sodium–calcium exchanger (NCX), a pivotal transmembrane transporter, mediates bidirectional transmembrane exchange of sodium and calcium ions across the plasma membrane. Aberrant NCX activity further disrupts cellular calcium homeostasis, aggravates excitotoxicity, and culminates in apoptotic or necrotic cell death [47].

### 2.6. Other Common Diseases

In addition to the previously mentioned common neurological diseases, Huntington’s disease is one of the more frequently encountered conditions in clinical practice. Its clinical manifestations include involuntary choreiform movements, cognitive decline, and psychiatric symptoms, with pathogenesis closely linked to mutations in the huntingtin gene [48]. Huntington’s disease is a hereditary neurodegenerative disease characterized by the abnormal expansion of CAG trinucleotide repeats (≥40 repeats) in the huntingtin gene. The primary pathological features of this condition consist of striatal atrophy, degeneration, and the loss of medium spiny neurons [49]. Genetic testing for CAG repeat enumeration is diagnostic, confirming mutation presence but not predicting symptom onset or trajectory [50]. Disease progression biomarkers—measuring mutant huntingtin protein aggregation, neurofilament light chain (NfL) elevation, or striatal atrophy rates—serve prognostic purposes, estimating time-to-symptom-onset and tracking neurodegeneration [51,52,53]. These distinct clinical purposes require separate validation frameworks: diagnostic tests demand analytical accuracy, whereas prognostic biomarkers require longitudinal predictive validity. The aberrant aggregation and impaired clearance of mHTT constitute the principal pathogenic mechanism underlying this disease. Under physiological conditions, mHTT species are modified by ubiquitin and subsequently recruit Siah-1-interacting protein to form complexes that regulate autophagosome assembly, thereby maintaining proteostasis in medium spiny neurons. When this clearance regulatory pathway is functionally compromised, mHTT accumulates intracellularly, resulting in the degeneration and death of striatal medium spiny neurons (MSNs), which ultimately contributes to the onset and progression of motor dysfunction in Huntington’s disease (Figure 2F) [54]. Beyond the canonical toxic gain-of-function induced by mHTT aggregation, emerging evidence has revealed that somatic expansion of the CAG repeat tract in the HTT gene exceeding 150 repeats transforms this relatively benign genetic variation into a pathogenic switch that directly triggers neuronal injury and cell death. This transition precipitates severe functional impairment and eventual neuronal death of neurons in the cerebral cortex and striatum [55], thereby propelling disease progression.

In summary, the hallmark of neurological diseases is the progressive dysfunction and death of neurons in specific anatomical regions [57]. These diseases are underpinned by a pathological foundation marked by the production of aberrant proteins that undergo conformational changes, form β-pleated sheet structures, and demonstrate a tendency for self-aggregation within neurons [58]. Simultaneously, oxidative stress, neuroinflammation, and mitochondrial dysfunction act as key pathogenic factors that impact these conditions [59].

## 3. Research Progress in Detection Methods

Biomarkers, as defined by the FDA-NIH BEST working group, refer to a characteristic that can be objectively measured and evaluated to indicate normal physiological processes, pathogenic processes, or biological responses to an exposure or therapeutic intervention [60]. The preceding sections have outlined the pathogenesis of various common neurological diseases alongside their pathological features at the molecular and cellular levels. It is precisely these alterations that provide the objective basis for disease detection. Surrounding the pathological processes, various detection technologies acquire disease-related information at multiple levels, including fluid molecules, extracellular vesicles, brain structural and functional imaging, and behavioral physiological signals. Achieving real-time, precise detection of neurological diseases is a crucial prerequisite for advancing targeted treatment. This objective relies upon the identification and analysis of specific biomarkers, thereby supporting precise detection and classification of different disease types. With breakthrough advances in biosensing technology, particularly the emergence of highly sensitive and specific sensors, detection platforms based on biosensors have become the central driving force of this field. Based on the physicochemical nature and transduction mechanisms of the captured signals, current cutting-edge research on biomarkers for neurological diseases primarily advances along four major technological paradigms: biosensing of biological molecules in body fluids (e.g., blood, CSF), analysis of surfaces and contents of nanoscale carriers such as exosomes, visualization of structure and function via multimodal imaging, and digital quantitative assessment based on wearable and behavioral data. These four paradigms together constitute a multi-level sensing network ranging from molecular to behavioral scales, and from in vitro assays to in vivo monitoring, providing a systematic technological solution for early warning, precise stratification, and dynamic monitoring of neurological diseases.

**Figure 3:**
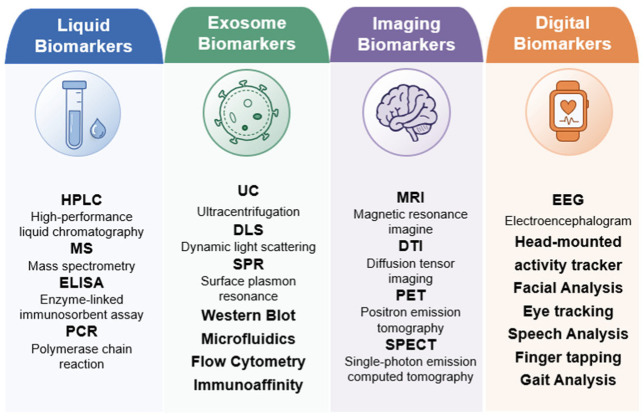
Summary of common detection methods. From left to right, respectively, are liquid biomarker detection utilizing molecular biochemical techniques to analyze protein-level changes in biofluids; exosomal biomarker detection applying separation and characterization methods to trace cellular origins; imaging biomarker detection leveraging modalities such as MRI and PET to visualize structural and functional brain abnormalities; and digital biomarker detection integrating wearable sensors with AI algorithms to enable dynamic, non-invasive monitoring of physiological and behavioral signals.

Common biomarkers and their corresponding detection approaches are summarized (Figure 3), and can be categorized as follows. Liquid biomarkers [61] are mainly derived from blood and CSF. The detection of molecules such as Aβ and tau, combined with techniques including MS [62], ELISA, and PCR [63], can reflect alterations at the molecular level. Exosome biomarkers [64] depend on separation technologies such as ultracentrifugation (UC) [65], immunoaffinity [66], and microfluidics [67], and achieve precise characterization by integrating DLS [68], SPR [69], and flow cytometry [70]. Imaging biomarkers visualize structural and functional changes in the brain via MRI [71], PET, SPECT [72], and other imaging approaches. Digital biomarkers [73] adopt multimodal sensors and artificial intelligence algorithms to analyze signals of eye movement, speech, locomotion, and sleep, realizing non-invasive, continuous, and dynamic monitoring. Biosensing technologies leveraging electrochemistry [74], optics [75], and advanced functional materials enable highly sensitive, rapid, and portable quantitative detection of liquid biopsy and exosomal biomarkers, offering robust technical support for molecular analysis. The clinical utility of biosensing paradigms is governed not by technical performance alone, but by the temporal demands of the diagnostic scenario. We should note that neurological biosensing operates across three distinct temporal regimes that fundamentally constrain biomarker and technology selection. These regimes differ not merely in duration, but in the physical and clinical imperatives they impose: a biomarker requiring 4 h laboratory processing is physically impossible for acute intervention regardless of analytical sensitivity, whereas a bedside test with 80% sensitivity may be preferable to a 99% sensitive laboratory assay in stroke because the 19% gain in accuracy cannot be realized within the therapeutic window.

For acute intervention (minutes to 6 h), therapeutic efficacy is exceedingly time-dependent: intravenous thrombolysis is effective within 4.5 h of onset, and endovascular thrombectomy within 24 h in select patients. Detection must prioritize speed and bedside deployability; non-contrast CT and CT perfusion are mandated to rapidly exclude hemorrhage and identify salvageable tissue [76,77]. In this regime, time-consuming liquid or exosomal assays are clinically irrelevant.

For pre-symptomatic detection (months to decades), early risk stratification operates on extended horizons. Alzheimer’s disease screening, for instance, targets a prodromal phase spanning 10–20 years. Plasma p-tau217 achieves high diagnostic accuracy but requires laboratory processing; this delay is trivial against the decades-long window for intervention [78,79]. Here, analytical sensitivity and specificity supersede speed.

For chronic monitoring (days to years), disease trajectory tracking occupies an intermediate position, requiring longitudinal consistency without demanding minute-by-minute resolution. Parkinson’s disease and multiple sclerosis exemplify this regime, driving interest in wearable platforms that balance accessibility with longitudinal stability [73].

These temporal regimes are not interchangeable. The same biosensing technology may be rejected in one context and embraced in another based not on technical merit alone, but on temporal appropriateness. The following sections examine each regime in detail, with disease-specific examples selected to illustrate how time constraints fundamentally shape biomarker and detection method selection.

### 3.1. Liquid Biomarker Detection Methods

Liquid biomarker detection, as a minimally invasive and dynamically informative characterization approach, has attracted considerable clinical attention [61,80]. Biological samples can be obtained from peripheral blood, CSF, and other accessible biofluids. By analyzing alterations in microRNA, peptides, small molecules, and protein abundances in these matrices, disease-associated differences can be identified, providing a sensitive strategy for screening neurodegenerative diseases [81,82,83]. Extensive efforts have been devoted to early diagnosis and longitudinal progression prediction [84]. As a core subset of liquid biopsy, CSF has long been regarded as a traditional and reliable source for neurological biomarker detection due to its direct contact with the central nervous system [85,86]. Representative biomarkers including β-amyloid 42 (Aβ42), Aβ40, total tau (t-tau), phosphorylated tau (p-tau), BACE1, and α-synuclein are well-established pathological indicators for major neurodegenerative disease. In particular, the Aβ42/Aβ40 ratio exhibits superior diagnostic performance compared with single Aβ42 measurement alone, enabling accurate discrimination of AD patients from healthy individuals [87,88,89,90,91,92]. Moreover, alterations in blood levels of p-tau, total tau (t-tau), BACE1, and α-synuclein may also provide valuable support for disease diagnosis and monitoring [83,93,94]. Among these biomarkers, p-tau has emerged as the gold standard blood biomarker for AD pathology [95]. Among the various p-tau isoforms, p-tau217 demonstrates superior diagnostic performance, exhibiting high specificity and stability for AD. It effectively discriminates AD from other neurodegenerative diseases, such as FTD and PD [89,96]. Notably, the ratio form of plasma p-tau217 on automated platforms outperforms both p-tau181 and p-tau231 [97]. Thus, while p-tau231 signals the earliest biological perturbation, p-tau217 provides the broadest diagnostic window. Plasma p-tau217 has recently been incorporated into the NIA-AA revised diagnostic criteria for AD (2023 update) [98], marking its transition from research tool to guideline-endorsed clinical assay. However, this endorsement is currently limited to symptomatic diagnostic confirmation rather than pre-symptomatic screening, reflecting the aforementioned blood–brain barrier limitations. However, the reliability of plasma biomarkers is contingent upon blood–brain barrier (BBB) integrity. In symptomatic AD patients, neuroinflammation disrupts the BBB, facilitating tau protein efflux into peripheral blood and enabling detectable plasma levels [99]. Conversely, during the pre-symptomatic phase, the BBB remains functionally intact, and neuron-derived tau cannot efficiently enter circulation; consequently, blood biomarker levels may remain normal despite active cerebral pathology [100]. This fundamentally constrains the utility of blood-based biomarkers for pre-symptomatic screening. Therefore, negative blood biomarker results in asymptomatic individuals cannot exclude underlying AD pathology, and imaging confirmation remains essential for pre-symptomatic screening. In addition to AD biomarkers, liquid biopsy strategies have been extended to other neurodegenerative diseases. For instance, Adil et al. [101] developed a label-free electrochemical immunosensor based on electrografted laser-induced graphene, which enabled direct detection of poly-glycine-proline dipeptide repeats in CSF with a LOD of 0.27 ng/mL, allowing effective discrimination between c9+ and non-c9 ALS patients. In epilepsy, blood-based biomarkers—particularly NfL—show promise for differential diagnosis and seizure characterization, though age adjustment and peripheral confounders limit clinical utility [102]. However, this sensor may face challenges when applied to blood samples due to the substantially lower concentrations of the target biomarkers and the more complex matrix interference present in peripheral blood. These limitations underscore the need for biosensing platforms that can achieve comparable sensitivity in minimally invasive biofluids.

With regard to detection tools, liquid biomarker analysis primarily relies on molecular-level biochemical techniques, including high-performance liquid chromatography (HPLC), mass spectrometry (MS), enzyme-linked immunosorbent assay (ELISA), and polymerase chain reaction (PCR). These approaches enable precise quantification of target molecules in biological fluids. Although CSF biomarkers exhibit the highest clinical specificity, their collection requires lumbar puncture, a procedure associated with high invasiveness and potential complications. This limitation restricts their applicability in large-scale population screening and long-term follow-up studies [103]. In contrast, minimally invasive biofluids such as blood are readily accessible; however, the concentrations of key neuropathological proteins in blood remain low due to the presence of the blood–brain barrier (BBB). Moreover, these protein levels are vulnerable to interference from peripheral physiological factors, resulting in insufficient specificity to meet the requirements of early diagnosis [85]. Consequently, such inherent bottlenecks highlight the urgent demand to develop high-performance biosensing methods and novel minimally invasive biomarkers that can accurately reflect core pathological processes within the central nervous system.

### 3.2. Exosomal Biomarker Detection Method

Exosomes, lipid bilayer microvesicles belonging to the family of extracellular vesicles (EVs), are emerging as a promising solution to the aforementioned bottlenecks [67,104]. Exosomal biomarkers remain predominantly in the preclinical proof-of-concept and early-phase clinical trial stage. No exosomal biomarker has achieved FDA approval or major guideline endorsement for routine neurological diagnosis [105]. However, exosomal biomarkers are not a homogeneous category. Plasma exosomes derive from multiple cellular sources—neurons, astrocytes, microglia, endothelial cells, and platelets—each carrying distinct molecular cargos that reflect different physiological or pathological states [106]. Neuron-derived exosomes (marked by L1CAM or NCAM) carry tau and synaptic proteins that indicate brain pathology [107]. Compounding this biological heterogeneity, current studies employ diverse isolation protocols—ultracentrifugation, precipitation, size-exclusion chromatography, and immunoaffinity capture—that differentially enrich for specific subpopulations and lack standardized definitions for exosomal purity or source specification [108]. This methodological inconsistency makes cross-study comparison problematic and severely limits the clinical applicability of published findings. This is not a minor technical detail but a fundamental implementation barrier: without source-specific isolation and validation, exosomal biomarkers will remain confined to research-stage investigations. Despite these challenges, exosomal biomarkers offer substantial promise for neurological disease detection. They specifically refer to functional biomolecular cargos encapsulated in exosomes, including proteins, nucleic acids, lipids, and metabolites, which have been widely recognized as ideal sensing targets for next-generation clinical biosensing platforms [105,109]. As a critical subtype of extracellular vesicles, exosomes can cross the BBB through multiple mechanisms and are widely distributed in various biological fluids. Their intrinsic molecular composition faithfully mirrors physiological and pathological alterations within the central nervous system, making them superior sensing analytes for early neurological disease screening [64,110]. Owing to their high structural stability, good biocompatibility, and easy accessibility in biofluids, exosomes maintain stable biochemical properties in complex body fluids, which greatly facilitates the development of stable, repeatable biosensing detection systems, making them ideal candidates for advanced liquid biopsy and biosensor-based clinical diagnosis [111,112].

To realize the effective utilization of exosomes, isolation and characterization techniques have continued to advance. Exosome isolation methods typically exploit their distinct biophysical or biochemical properties and can be broadly categorized into approaches such as UC [65] and immunoprecipitation [66]. In addition, recent developments in microfluidic and nanomaterial-based technologies have introduced innovative strategies to enhance exosome isolation efficiency and purity [113,114]. Microfluidic technology, in particular, provides novel avenues for efficient exosome isolation, enabling high-purity separation directly from complex biological fluids. Owing to their uniform size distribution during collection, exosomes can be effectively separated from other nanoscale particles using microfluidic platforms, providing high-quality samples for subsequent biosensor signal acquisition [115]. Moreover, microfluidic channels are well suited for continuous and high-throughput exosome isolation, highlighting their potential for future clinical applications [116]. Following isolation, comprehensive characterization of exosomes is required to confirm their identity and to distinguish specific subpopulations. Over the past decades, a range of characterization techniques has been developed based on exosomal biological features, including ELISA, dynamic light scattering (DLS), immunoblotting, flow cytometry, and surface plasmon resonance (SPR) imaging. Among these technologies, SPR represents one of the most representative label-free optical biosensing platforms, which enables real-time monitoring of molecular binding events by detecting subtle refractive index and mass changes on the plasmonic interface. Owing to its ultra-high sensitivity, excellent specificity, and label-free detection capability, SPR biosensors have been widely employed for exosomal phenotype identification, subpopulation differentiation, and biomolecular interaction analysis [117]. Besides optical SPR sensing, electrochemical biosensors have become another research hotspot in exosome detection due to their low cost, miniaturization convenience, and high quantitative accuracy. Typical high-performance exosome biosensors generally achieve a low detection limit at the order of 10^4^ particles·mL^−1^, with a wide linear detection range covering 10^3^–10^7^ particles·mL^−1^, enabling trace-level detection of pathological exosomes in early disease stages [118,119]. Xie et al. [120] constructed an electrochemical biosensor using MR-1 bacteria and Cu-TCPP MOF for exosome detection, with a linear range of 1.38 × 10^3^–1.38 × 10^7^ particles·mL^−1^ and an LOD of 659 particles·mL^−1^. However, this method depends on costly aptamers and lacks antifouling ability in complex biofluids. Despite these advances, liquid and exosomal biomarkers provide only a molecular-level snapshot of neurological diseases. Individual molecular markers remain insufficient to comprehensively capture the macro-level effects of disease on brain regional structure, functional networks, and patient behavior [121]. To supplement in situ spatial localization of lesion distribution, accurate assessment of disease severity, and intuitive characterization of neural functional degeneration, research inevitably promotes the combination of biosensing with non-invasive imaging detection and multidimensional behavioral evaluation methods [122].

### 3.3. Imaging Biomarker Detection Method

Given the lack of spatial anatomical and locational information provided by conventional molecular biomarkers, imaging biomarkers have emerged as an indispensable complementary sensing strategy. The European Imaging Biomarker Alliance for Radiology (EIBALL) defines imaging biomarkers as “image features associated with patient diagnosis”, which can be quantitatively extracted and analyzed as visual sensing signals for auxiliary disease identification.

Imaging examinations serve as macroscopic diagnostic tools for evaluating tissue structure and function. Key modalities include magnetic resonance imaging (MRI), functional MRI (fMRI), diffusion tensor imaging (DTI), positron emission tomography (PET), and single-photon emission computed tomography (SPECT), all of which enable direct visualization of cerebral structural, functional, and metabolic alterations [122]. As a vital branch of in vivo biosensing, molecular imaging (e.g., DAT-SPECT) achieves high diagnostic accuracy (sensitivity up to 100% for confirmed PD) in visualizing nigrostriatal dopaminergic loss. However, its utility in population-level biosensing remains limited by high costs, ionizing radiation, and the inability to differentiate between different neurodegenerative parkinsonian syndromes [123]. Ultra-high-field MRI allows detailed observation of deep brain regions such as the substantia nigra, thereby revealing pathological lesions within this area. DTI primarily yields two key parameters: the mean diffusion coefficient and fractional anisotropy, which reflect microstructural integrity of white matter. PET and SPECT tracers are widely used for dopamine transporter (DAT) imaging to investigate striatal dopaminergic terminal function, enabling visualization of cellular activity and molecular processes in the living human brain [124]. The standard workflow of imaging biomarker detection typically involves image acquisition, segmentation of regions of interest (ROIs), extraction and filtering of imaging features, classification and model construction, database establishment and sharing, and personalized data analysis. Quantitative imaging biomarkers benefit from standardized protocols for multi-center deployment to improve reproducibility across scanners and sites, whereas qualitative visual assessment, while clinically intuitive, exhibits higher inter-rater variability and is less suitable for longitudinal monitoring where consistent measurement thresholds are preferred, forming a complete closed-loop imaging sensing and intelligent diagnosis system. Ultra-high-field MRI refers to magnetic resonance techniques operating at field strengths of 7 T or higher [71]. Taking 7 T MRI as an example, this technology enables high-resolution visualization of hippocampal subregion integrity and the locus coeruleus. The locus coeruleus is the primary source of noradrenaline, a neuromodulatory neurotransmitter that regulates cognitive functions such as arousal and attention. Evidence suggests that the contrast-to-noise ratio within the locus coeruleus, as assessed by ultra-high-field MRI, can predict the responsiveness of patients with PD or progressive supranuclear palsy to noradrenergic therapies [125,126,127]. In addition, arterial spin labeling (ASL) MRI can be used to quantify cerebral blood flow, while quantitative susceptibility mapping (QSM) enables estimation of cerebral oxygenation, thereby facilitating the evaluation of microvascular dysfunction associated with early-stage AD [128]. Kim et al. [129] found that their developed gadolinium-labeled aptamer MRI contrast agent (GdDOTA-ob5) can specifically target oligomeric amyloid-β in AD mouse models, achieving sensitive in vivo imaging of early pathological deposits, and this probe exhibits higher relaxivity than clinical contrast agents. The ILAE classification framework [130] establishes a three-level diagnostic hierarchy for epilepsy: seizure type, epilepsy type, and epilepsy syndrome, operationalized through seizure semiology, EEG, and neuroimaging. Notably, quantitative EEG biomarkers and blood-based biomarkers are not currently incorporated into this framework and remain outside ILAE-endorsed guidelines. In epilepsy, quantitative EEG biomarkers—particularly high-frequency oscillations (HFOs, 80–500 Hz)—enable localization of epileptogenic tissue, though intracranial recordings remain the gold standard for HFO detection [131,132].

Despite the authoritative role of imaging biosensing in disease diagnosis, staging and pathological mechanism analysis, and the complementary combination of conventional liquid biosensing and imaging sensing to assess normal biological processes, pathological alterations, and pharmacological responses in neurological populations, this modality still presents obvious inherent limitations for clinical translational application. From the perspective of biosensing popularization, ultra-high-field MRI and PET are expensive and are highly dependent on professional equipment and technical personnel; the relatively long detection time and high examination cost limit their large-scale application in community early screening. Moreover, imaging detection cannot support long-term, continuous home-based dynamic monitoring, which presents substantial challenges for routine early screening and repetitive evaluation of therapeutic efficacy [91,133].

### 3.4. Digital Biomarker Detection Method

To address the high costs and limited temporal continuity associated with imaging-based assessments, researchers have increasingly focused on non-invasive, wearable, biosensor-integrated and real-time digital biomarkers for nervous system diseases [73]. Digital biomarkers are specifically designed for remote neurocognitive data acquisition and artificial-intelligence-driven intelligent sensing analysis. They primarily consist of digital data generated through the continuous collection and quantitative analysis of physiological signals using biosensors or portable digital devices [134,135]. At present, no curative treatment exists to halt or reverse the progression of most neurological diseases. As physiological signals dynamically fluctuate during daily activities, the targeted analysis of digital biomarkers enables real-time monitoring and early preclinical detection, highlighting their growing importance in disease screening and longitudinal assessment [136]. When combined with high-performance biosensors, digital biomarkers enable continuous, real-time, and non-invasive monitoring of neurodegenerative diseases, serving as a valuable complement to traditional biomarkers [91].

**Figure 4:**
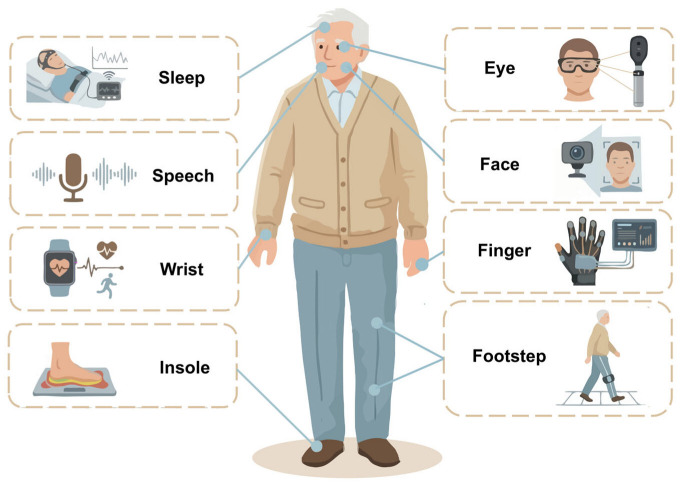
Schematic overview of digital biomarker-related devices for neurological disease assessment. Wearable devices can be attached to different body regions (e.g., wrist-worn smartwatches, sleep and voice monitors, and smart insoles), while complementary systems are used to evaluate motor and non-motor symptoms across multiple anatomical sites, including finger motion sensors, eye- and facial-analysis tools, and gait monitoring systems.

To date, a diverse range of digital methodologies has been developed for the detection of neurological diseases. Wrist-worn or head-mounted activity monitors for sleep assessment, ocular imaging, and eye-tracking combined with facial analysis, voice analysis, and finger-tapping tasks are widely employed in digital biomarker research [137]. These devices can be worn on different parts of the body or deployed within sensor- equipped environments, enabling flexible and scalable data collection (Figure 4). Digital biomarker acquisition can be either active, requiring users to perform designated tasks, or passive, in which data are continuously collected without explicit user engagement. The latter approach minimizes interference with daily activities and allows for long-term, high-frequency data acquisition [138].

Digital biomarkers vary by disease context. In AD, cognitive impairment primarily manifests as memory decline, language dysfunction, and spatial disorientation. Wearable devices for reaction time measurement, sleep quality tracking, and physical activity monitoring offer non-invasive cognitive assessment tools [127,139]. Wrist-worn activity monitors track sleep and physical activity, while GPS and smartphone-based sensors capture gait patterns, speech characteristics, and typing dynamics. In PD, motor impairments primarily manifest as resting tremor, muscle rigidity, and bradykinesia. Eye-tracking technologies have been widely applied to distinguish PD patients from healthy controls [91,140,141,142,143,144,145]. More recently, Vásquez-Correa et al. [146] proposed a deep learning system integrating multimodal signals—speech, handwriting, and gait—for PD assessment, achieving 97.3% classification accuracy when all three modalities were fused. For Parkinson’s disease, the MDS clinical diagnostic criteria establish motor parkinsonism as the core diagnostic feature, assessed through the MDS-UPDRS [147]. Wearable sensor metrics and digital biomarkers are not currently incorporated into these criteria and remain in the research validation stage. In epilepsy, digital biomarkers from wearable devices offer continuous, non-invasive seizure monitoring. Multi-sensor platforms integrating accelerometry enable real-time seizure detection, though performance varies across patients and regulatory approval for clinical-grade monitors remains limited [148,149].

Despite these promising technical performances, digital biomarkers, including wearable sensor platforms and AI-driven algorithms, remain in the research validation and early clinical implementation stage, with no FDA-approved standalone diagnostic tool for neurological disease yet available [150]. However, these metrics require cautious interpretation: the 97.3% accuracy reflects within-cohort cross-validation under optimal laboratory conditions, where distributional biases and site-specific noise systematically inflate apparent performance [151]. Prospective independent validation routinely yields lower performance, yet this critical step is frequently omitted. The gap between research efficacy and real-world effectiveness is substantial: controlled settings achieve high accuracy, whereas home-based deployment degrades markedly due to non-compliance, environmental variability, and signal dropout [152,153]. In the mPower study, substantial participant attrition occurred over time, with compliance dependent on proactive engagement [154]. Without sustained support, patients discontinue use due to discomfort and technical difficulties, rendering algorithmic accuracy clinically meaningless. Digital biomarker validation must therefore mandate adherence metrics and real-world noise tolerance as co-primary endpoints alongside technical accuracy.

Language impairment is a prominent feature of the early stages of ALS, as progressive bulbar motor degeneration ultimately leads to deficits in speech and swallowing functions [155]. Speech science traditionally regards the bulbar system as part of a broader speech production network comprising four interrelated subsystems: respiration, phonation, articulation, and resonance [156]. These subsystems provide more sensitive measures than current standard clinical assessments for detecting subclinical changes in bulbar motor performance [157]. Evaluation of speech motor performance—particularly parameters such as tongue movement velocity—may therefore be highly suitable for early disease detection and longitudinal monitoring. Speech-based biomarkers also offer notable advantages in accessibility, as they can be readily collected using portable devices, including smartphones. Consequently, multidisciplinary research efforts are increasingly focused on developing advanced speech movement tracking hardware and intelligent algorithms to identify abnormal speech and vocal patterns within large-scale datasets [158]. Within the digital biomarker framework, artificial intelligence algorithms have begun to be explored for the automated recognition of complex data patterns and the integration of multidimensional physiological signals, further improving the accuracy and reliability of digital biomarker-based disease diagnosis.

## 4. Comparison and Evaluation of Detection Methods

**Figure 5:**
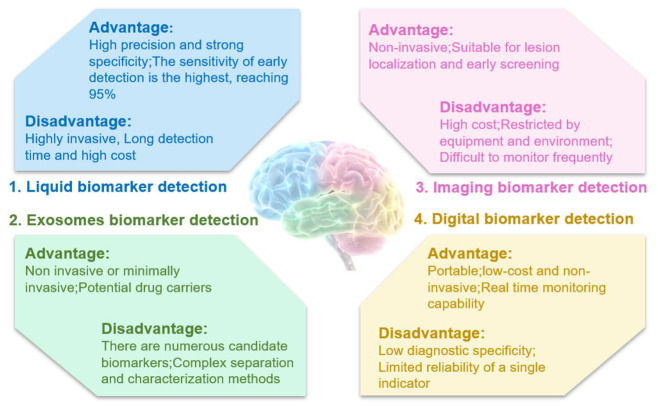
Advantages and disadvantages of commonly used analytical methods: a brief review.

The diagnostic performance, clinical maturity, cost-effectiveness, and practical applicability of different biomarker types vary substantially in the detection of neurological disease. Liquid biomarkers, exosomes, medical imaging, and digital biomarkers each exhibit distinct characteristics in terms of sensitivity, detection scope, and relative advantages and limitations. To facilitate a comparative understanding of these four approaches, a multimodal biomarker detection framework is presented in Figure 5. A detailed comparison of their sensitivity, detection range, and key strengths and limitations is summarized in Table 1.

### 4.1. Evaluation of Liquid Biomarker Detection Methods

Given the relatively stable microenvironment and molecular composition of CSF, any significant deviation from its normal physiological range may serve as a specific and sensitive early warning indicator for nervous system diseases. Among diverse individual fluid biomarkers, p-tau217 demonstrates outstanding and superior diagnostic performance in the targeted identification of AD, with a diagnostic sensitivity of 0.95 and specificity of 0.94: its core diagnostic parameters significantly outperform other conventional liquid biomarkers including β-amyloid, BACE1, and total tau (t-tau) [159]. However, from the perspective of clinical translation and practical application maturity, CSF biomarker detection relies on lumbar puncture for sample acquisition. This invasive procedure carries clinical risks including central nervous system herniation, infection, and hemorrhage, while also causing obvious physical discomfort and reducing patient compliance, thereby limiting its widespread clinical adoption [133]. At the cost-effectiveness and detection efficiency levels, existing mainstream detection technologies such as MS, ELISA, Western blotting, and HPLC are associated with high instrument and reagent costs, as well as cumbersome, time-consuming sample preparation and pretreatment processes, which substantially increase the economic and time costs of clinical testing. From the perspective of diagnostic accuracy and applicability, liquid biomarkers are intricately associated with diverse physiological mechanisms. Reliance on single or limited biomarkers for detection readily yields false-positive or false-negative results, rendering precise disease diagnosis challenging. Multiplex biomarker panels are therefore required to enhance diagnostic accuracy [160,161]. Quantitative evaluation reveals the insufficiency of single-biomarker deployment. Palmqvist et al. [162] demonstrated that plasma p-tau217 as a stand-alone test achieved a positive predictive value (PPV) of 79% (95% CI, 74–84) in a population with 33.3% amyloid positivity. However, this performance does not translate to screening settings where prevalence is lower. Assuming 95% sensitivity, a screening population with 10% prevalence yields a PPV of only 74%, meaning 26% of positive tests are false alarms. At 5% prevalence, PPV drops further to 62%, with false positives outnumbering true positives nearly 2:1. For comparison, a Chinese community screening cohort with age-specific thresholds targeting PPV of 0.70 reported overall p-tau217 positivity of 11.8%, with false-positive rates increasing in younger cohorts [163]. This would generate substantial unnecessary referrals for confirmatory testing.

Multimarker detection substantially improves performance. The combination of plasma p-tau217 and Aβ42/40 achieved an AUC of 0.949 (95% CI, 0.929–0.970), significantly higher than p-tau217 alone [164]. The PrecivityAD2 algorithm combining p-tau217 and Aβ42/40 yielded an AUC of 0.94, with 88% agreement with amyloid PET [165]. A two-step workflow (plasma p-tau217 followed by confirmatory PET or CSF) increased PPV from 79% to 91% (95% CI, 86–95) [162]. These findings support multimarker strategies as clinically advantageous for reliable population-level screening.

Recent clinical implementation frameworks support this transition. The Swedish BioFINDER study has established longitudinal protocols integrating plasma p-tau and other complementary biomarkers for clinical decision-making [166]. The US AHEAD 3–45 study, a secondary prevention trial targeting preclinical and early preclinical AD (intermediate amyloid stages), is the first to employ plasma biomarkers to accelerate screening and substantially reduce PET scan burden. These data establish that multimarker strategies are not merely theoretically advantageous but essential for clinically deployable screening. Single biomarkers with acceptable performance in high-prevalence symptomatic cohorts become clinically problematic in low-prevalence screening populations due to unacceptably high false-positive rates. Until cost-effectiveness analyses directly compare single-marker, multimarker, and imaging-first pathways across diverse healthcare systems, validated multimarker algorithms should be prioritized to minimize false-positive-driven downstream costs and patient burden.

### 4.2. Evaluation of Exosomal Biomarker Detection Methods

Exosomes, comprising a lipid bilayer that effectively protects their cargo from immune clearance and enzymatic degradation, exhibit high stability in bodily fluids. Their widespread presence in easily accessible fluids, including saliva, urine, sweat, respiratory secretions, and tears, provides non-invasive and convenient opportunities for disease detection, potentially serving as an alternative to invasive CSF sampling in certain clinical contexts [167,168]. From a comprehensive application perspective, within tissues, exosomes can be engineered to deliver targeted molecules, thereby reducing systemic toxicity and enhancing drug safety and stability. In addition, exosomes can partially evade phagocytosis and macrophage-mediated degradation, prolonging in vivo circulation, enhancing biological activity, and achieving therapeutic effects [169]. Exosome-based detection approaches thus hold substantial theoretical significance and translational potential. Nonetheless, several challenges remain for clinical implementation. These include their relatively short in vivo circulation half-life and susceptibility to clearance, factors that greatly restrict their clinical utility and limit therapeutic efficacy. Although strategies such as surface modification and encapsulation carriers have been proposed, comprehensive evaluation of stability and long-term safety is still required [170,171]. Furthermore, the lack of standardized regulatory frameworks complicates clinical translation, necessitating ongoing improvements in production protocols, quality control, and regulatory compliance [172]. In summary, compared with other biomarker detection methods, this dual capacity for diagnosis and therapy positions exosomes as a compelling exemplar of the diagnostic–therapeutic integration paradigm in the evolution of biomarker technologies.

### 4.3. Evaluation of Imaging Biomarker Detection Methods

Brain structural and functional imaging techniques, including MRI, PET, and SPECT, provide detailed morphological, metabolic, and functional information, enhancing early diagnosis of neurological diseases [173]. These modalities are non-invasive and reproducible, representing essential tools for routine clinical diagnosis and disease progression assessment [174]. In terms of diagnostic performance, a meta-analysis of five studies using 7T MRI for PD demonstrated that loss of the “feathering sign” achieved 99% sensitivity and 92% specificity in distinguishing PD patients from healthy controls [122]. However, the clinical application of imaging biomarkers faces substantial practical constraints. Ultra-high-field 7T MRI systems in the United States require an initial investment of approximately $10 million, with a single standardized brain structural scan taking up to 75 min [175]. Conventional brain FDG-PET costs $1358 on average [176]. These costs, combined with limited scanner availability and dependence on specialized equipment typically confined to hospitals or research laboratories, render population-level screening economically unfeasible. Furthermore, imaging modalities are poorly suited for bedside, home-based, or long-term dynamic continuous monitoring, exposing inherent limitations in early disease warning and longitudinal follow-up.

### 4.4. Evaluation of Digital Biomarker Detection Method

Digital bioanalytical methods, which correlate with cognitive and motor functions, provide novel and objective evidence for the diagnosis of neurodegenerative diseases. The development of portable and compact measurement devices enables continuous monitoring and documentation of patient status, surpassing the traditional physical limitations of clinical settings [177]. From the perspective of prospective application, in terms of clinical maturity, digital biomarkers remain in the developmental stage; however, they exhibit high clinical accessibility and demonstrate substantial potential for early disease warning and dynamic monitoring. Nonetheless, it is worth noting that neurological diseases are multidimensional and multifactorial syndromes that cannot be fully characterized by a single digital metric. Integrating multiple digital biomarkers into a unified diagnostic framework significantly improves diagnostic accuracy and reliability [134]. In comparison with other detection methods, regarding cost-effectiveness, this detection modality, relying on portable devices, demonstrates favorable overall economic viability. Even with the above advantages, practical implementation of digital bioanalysis, however, remains challenging. These challenges include extracting meaningful signals from substantial background noise, the absence of standardized data validation protocols, low patient adherence to long-term device usage, and insufficient participant retention in remote studies. All these bottlenecks seriously restrict the clinical popularization and large-scale application of digital biomarkers. Moreover, mature technologies to safeguard patient privacy are still limited [138,153].

However, the cited classification accuracies for digital biomarkers derive from controlled research settings with compliant participants. The gap between experimental algorithm performance and real-world effectiveness—where patient adherence, environmental noise, and device variability substantially degrade accuracy—is not adequately captured by laboratory metrics. Among the four detection modalities, liquid biopsy biosensing achieves high specificity and an ultra-low detection limit of 0.1 pg/mL (Aβ_1–42_) within several minutes [178,179], whereas its clinical translation is severely constrained by invasive sampling procedures and complicated sample pretreatment with a lengthy turnaround time. Exosome-based biosensing exhibits excellent stability and non-invasive characteristics with a detectable sensitivity of 1.2 × 10^2^ ± 0.16 × 10^2^ particles/mL and rapid response capability [180], though it is hindered by the absence of unified standardized isolation and purification protocols, restricting its large-scale clinical promotion. Imaging biosensing (Amyloid-PET) allows non-invasive visualization with a diagnostic sensitivity of 96% for neuropathologically confirmed AD [181]. Amyloid PET imaging represents a mature, FDA-approved diagnostic modality with three clinically approved radiotracers ([18F] florbetapir, 2012; [18F] flutemetamol, 2013; [18F] florbetaben, 2014), all approved to estimate amyloid neuritic plaque density in adults with cognitive impairment being evaluated for AD. These approvals were based on pivotal PET-to-autopsy validation studies. In 2020, [18F] flortaucipir (Tauvid) became the first FDA-approved tau PET tracer. Updated appropriate use criteria for amyloid and tau PET have been published by the Alzheimer’s Association and Society for Nuclear Medicine and Molecular Imaging Workgroup, reflecting the established clinical utility of these imaging biomarkers [182]. By contrast, wearable-based digital biosensing delivers portable real-time detection within minutes, with an accuracy exceeding 90% under controlled conditions, yet it still faces inherent challenges such as unavoidable signal noise and imperfect unified technical standards for clinical implementation. Furthermore, the sensitivities exceeding 90% reported for AI and digital biomarker studies require cautious interpretation: these metrics often derive from single-center, retrospective cohorts with homogeneous patient populations, raising substantial concerns regarding selection bias and overfitting. True external validation—particularly across diverse populations, device platforms, and clinical settings—remains scarce, compromising reproducibility and multi-center generalizability. Regulatory certification, clinical responsibility, and algorithmic update protocols remain largely unaddressed. Transparent reporting frameworks (TRIPOD+AI, PROBAST+AI, DECIDE-AI) are essential, yet their adoption in neurological biosensing research remains limited [183,184,185]. These methodologic and governance gaps represent substantial barriers to clinical adoption beyond technical proof-of-concept. Collectively, each modality presents distinct strengths and inherent technical bottlenecks in terms of sensitivity, limit of detection, response efficiency, and practical applicability, highlighting the urgent need for further in-depth exploration of sensing mechanisms and optimization of technical specifications to address realistic clinical constraints in future research. Biosensing technology is deeply integrated into the four major detection modalities of liquid biopsy, exosomes, imaging, and digital biomarkers, enabling rapid biomarker detection for disease prevention and precision diagnostics. Biosensing technology is advancing toward flexibility [186], intelligence [187], and miniaturization [188]. Commonly employed sensing materials include traditional metallic materials, organic conductive materials, and gel-based materials, among which Gallium-based liquid metal, leveraging its high electrical conductivity, exceptional flexibility, and favorable biocompatibility, has emerged as a functional material for biosensing applications [189]. The empowered biosensing system not only fabricates highly compatible 2D/3D [190] sensing interfaces for liquid biopsy, enabling ultrasensitive quantitative detection of low-concentration neural markers in blood and CSF, but also optimizes biosensing performance for microfluidic isolation and characterization of exosomes. Simultaneously, it serves as the core biosensing signal acquisition module in wearable devices for digital detection, and can be integrated with imaging technologies to construct unified biosensing systems, expanding the dimensions of neural electrophysiological signal detection. These advances collectively enhance the sensing performance of all four detection modalities in terms of sensitivity, flexibility, and biocompatibility. Nevertheless, the large-scale clinical translation of such integrated biosensing platforms still faces challenges such as cost control and long-term stability verification, which require further in-depth exploration.

From the perspectives of detection mode, cost, test duration, and clinical application, liquid biomarkers adopt automatic immunoassay with moderate cost, taking about 4–6 h and widely used in hospitals after FDA approval. Exosomal biomarkers need complex separation, costing more and taking roughly 6–12 h, which are only studied in labs without mature clinical schemes. PET/MRI biomarkers are costly and need about 2–4 h for full inspection; they are guideline-recommended but only available in professional imaging centers. Digital biomarkers collect signals via wearable devices with low cost and instant results, which are mostly trialed for home monitoring rather than formal diagnosis.

**Table 1:** Comparison of Detection Performances of Common Testing Methods.

Detection Method	LOD	Sensitivity	Advantages	Disadvantages	Ref.
Liquid Biomarker Detection	LOD: 0.0015 pg (p-tau217)	~90% (p-tau217)	The Simoa plasma p-tau217 assay offers high diagnostic accuracy, full automation, and high analytical sensitivity as its core advantages, making it suitable for routine clinical application and triage screening.	A relatively high intermediate zone proportion, performance variability across cohorts, the impact of comorbidities, and limitations in racial/ethnic data represent concerns that require attention during real-world deployment, particularly necessitating cautious interpretation in diverse populations.	[191]
Exosomal Biomarker Detection	LOD: 10 fg/mL (Aβ1-42)	~95.0% (Aβ1–42)	Less invasive, easier to implement, and more accessible for diagnosis than the currently validated cerebrospinal fluid assays and the biomarker triad in PET imaging.	The sample size detected by this technology in the present study was limited. Considering subsequent preclinical applications, a large number of clinical samples is required to establish reliable detection standards.	[192]
Imaging Biomarker Detection	LOD: 7.3 pg/mL (p-tau217)	95% (p-tau217)	The assay offers ultra-high accuracy, large effect size, and ultra-sensitive technology as its core advantages.	limited sample size, high cost, lack of head-to-head comparisons, unclear impact of comorbidities and race, and insufficient longitudinal data represent critical issues that need to be addressed before widespread clinical deployment.	[193]
Digital Biomarker Detection	LOD: 980 fg mL^−1^ (p-tau 181)	100% classification accuracy (p-tau 181)	The classifier achieved 100% classification accuracy with no false positives or false negatives.	The lack of real-world validation, unconfirmed long-term stability, unclear regulatory pathways, and challenges in scalable production represent critical barriers before clinical deployment.	[194]

## 5. Conclusions and Future Perspectives

**Figure 6:**
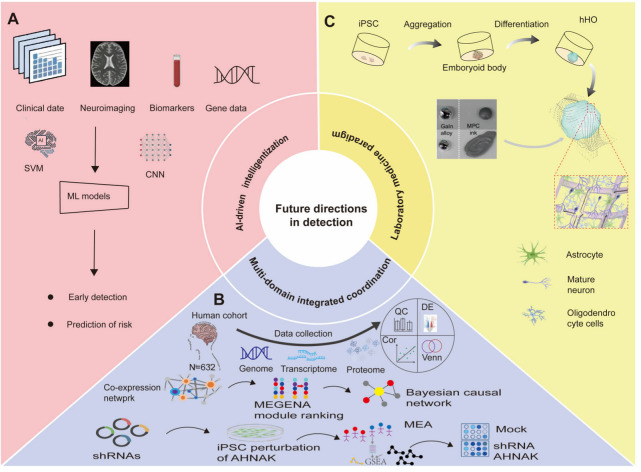
Principal future development directions for neurological disease detection technologies. (**A**). AI-driven intelligent assisted diagnosis for Parkinson’s disease, integrating multi-source data and multiple machine learning algorithms to achieve early screening, differential diagnosis, and risk prediction; (**B**). Multi-domain synergistic integration for screening AD pathogenic protein networks. Adapted from Wang et al. [195], CC BY 4.0; (**C**). Paradigm shift in laboratory medicine, schematic illustration of three-dimensional liquid metal-based neural interfaces for human hippocampal organoids. Adapted from Wu et al. [196], CC BY 4.0.

This review summarizes recent advancements in bio-detection methodologies for common neurological diseases, like AD, PD, epilepsy, and stroke, outlining the types, mechanisms, and disease-specific bio-detection strategies. Based on the classification of detection substrates and signal transduction mechanisms, four main directions of bio-detection research were analyzed, including liquid, exosomal, imaging, and digital biomarker detection, with the advantages of biosensing technology summarized in these directions. According to the current development trend, emerging biosensing paradigms are converging on three strategic frontiers: multimodal sensor fusion for comprehensive biomarker profiling, single-molecule precision for ultrasensitive detection thresholds, and continuous temporal monitoring for dynamic disease trajectory tracking. By harnessing AI-enabled pattern recognition, cross-modal biosensor synergy, and temporally resolved biomarker tracking, these integrated platforms will operationalize precision neurology—enabling early risk stratification, disease trajectory forecasting, and treatment response prediction, summarized in Figure 6.

### 5.1. AI-Driven Intelligent Upgrading

The integration of artificial intelligence (AI) and machine learning (ML) with biosensing platforms represents a transformative inflection point in neurological diagnostics. In particular, ML technologies greatly improve diagnostic precision and early biomarker detection abilities, offering more objective assessment methods [197]. In the domain of precise diagnosis for AD, multimodal data fusion strategies have been demonstrated to possess substantial applied value. Studies have shown that integrating AI with classical neurological biomarker assessments to construct optimal decision spaces enables precise differentiation among individuals with AD, mild cognitive impairment, and normal cognition [198]. This demonstrates that cross-modal biomarkers integrated with AI yield synergistic gains in diagnostic accuracy beyond unimodal approaches. Additionally, a study utilized support vector machine algorithms, as one ML method, to develop classifiers that effectively differentiate between cognitively normal, cognitively impaired, and severely cognitively impaired populations based exclusively on test performance, achieving high classification accuracy. The classifier exhibited optimal predictive performance for AD with accuracy, sensitivity, and specificity recorded at 97.74 ± 5.78%, 97.99 ± 5.78%, and 96.08 ± 4.33%, respectively [199]. In a differential diagnosis of dementia with Lewy bodies and PD, algorithms such as logistic regression, K-nearest neighbors (K-NN), and support vector machines (SVMs) have shown considerable promise. Notably, the K-NN classification model attained an overall accuracy of 91.2% [200]. The integration of edge-AI algorithms with electrochemical or optical transduction systems enhances diagnostic performance through real-time signal denoising, adaptive calibration, and predictive anomaly detection, thereby establishing the technical prerequisites for point-of-care neurological screening and longitudinal disease monitoring. However, the scalability of such AI-biosensor systems depends on robust infrastructure for multi-center data validation and standardized biobanking protocols—a challenge that necessitates federated learning architectures and privacy-preserving computational frameworks to overcome legal and ethical barriers to large-scale data sharing. It is important to recognize that although data and sample sharing are essential for advancing large-scale biomarker research and development, adherence to complex legal and ethical requirements is imperative when sharing patient data. Progress toward standardized, ethically compliant data infrastructures will determine the scalability of intelligent biosensing from proof-of-concept demonstrations to clinically validated diagnostic platforms.

### 5.2. Multi-Domain Synergistic Integration

Multimodal data integration is essential for enhancing diagnostic efficacy. The deep fusion of various multimodal information, such as MRI, PET, clinical data, and genetic data, through cross-attention mechanisms minimizes inter-modal discrepancies and effectively integrates features across modalities. This approach significantly enhances diagnostic performance [201]. Simultaneously, a multi-domain synergistic model that integrates “molecular-level + structural–functional + behavioral phenotypic” dimensions allows for a more comprehensive understanding of disease pathological characteristics, thereby offering more substantial evidence for early diagnosis and monitoring of disease progression. Global-scale collaborative initiatives have illustrated the benefits of extensive research. The Global Neurodegenerative Proteomics Consortium has created comprehensive clinical datasets encompassing AD, PD, FTD, and ALS, with open access implemented in July 2025, providing crucial data support for cross-regional, large-scale investigations [202]. In terms of algorithm optimization, deep learning models employing hierarchical extraction of key features enable more comprehensive information mining from multimodal data, thereby further enhancing the accuracy and stability of AD diagnosis [203]. Furthermore, the integration of single-cell multi-omics, including epigenetics, with dietary intervention strategies has opened new avenues for identifying innovative biomarkers and therapeutic approaches for neurological diseases [204]. Regarding multimodal system expansion, wearable biosensing technologies enable the incorporation of dynamic physiological and biomolecular signals into synergistic analysis, effectively compensating for the cumbersome acquisition and limited spatiotemporal coverage of conventional imaging data, thereby achieving more comprehensive, real-time, and continuous portable dynamic monitoring of disease onset and progression. Future endeavors must further strengthen cross-technology, cross-institutional, and cross-regional collaborative partnerships, establish standardized data platforms and technical specifications, and promote deep integration and clinical translation of multi-domain technologies.

### 5.3. Paradigm Change in Laboratory Medicine

The paradigm of laboratory medicine is changing from population-based universal reference intervals to individualized, data-driven, precision-stratified reference intervals, which enhances diagnostic accuracy at the individual level [205]. To achieve individualized, precision-oriented differential diagnosis with enhanced accuracy, these demands have propelled the emergence of novel detection technologies, including biosensor-based assays, wearable flexible sensors, and in situ rapid analysis. These technologies not only provide critical support for non-invasive, real-time, and dynamic detection of neurological diseases, but also promote the progressive advancement of bodily fluid biomarker detection toward high sensitivity, high-throughput, and point-of-care products. For instance, Zhao et al. [206] developed a fully integrated, wearable, sweat-sensing patch incorporating microfluidic collection, multi-electrode sensing, and wireless transmission modules, enabling non-invasive, real-time online detection of L-dopa and other PD-related biomarkers in sweat, with clinical validation establishing its efficacy for early screening and dynamic monitoring of PD. Current advancements in neurological disease detection technology are progressing towards non-invasiveness, portability, flexibility, and real-time capability, with liquid metal playing a pivotal role as a key material in flexible electronics and sensing domains. Wu et al. [196] integrated liquid metal–polymer conductor (MPC) mesh neural interfaces with human hippocampal organoids, successfully achieving non-invasive detection of neural activity. Concurrently, existing studies have confirmed that gallium-based liquid metals demonstrate excellent biocompatibility with human blood and serum, exerting minimal impact on physiological blood systems [207], thereby establishing a safety foundation for their further application in biosensor detection. With the progressive maturation of technologies and materials, disease diagnosis has further extended toward the molecular level. Single-cell extracellular vesicles were instantly detected to reveal that membrane-bound α-synuclein acts as an ultra-early biomarker for AD, achieving area under the curve values of 0.93 and 0.95 for prodromal and clinical stage diagnoses, respectively. This provides robust evidence for personalized early precision intervention [208].

In summary, future neurological diagnostics will transcend the spatiotemporal confines of hospital-centric care, extending toward decentralized, home-based monitoring architectures that enable continuous disease trajectory management. This evolution hinges on the convergence of three technological pillars: wearable biosensing platforms for real-time biochemical surveillance, liquid-metal-enabled flexible electronics for seamless physiological integration, and AI-driven analytics for predictive risk stratification. By embedding preventive capabilities into daily life, these integrated systems will catalyze the clinical paradigm shift from episodic intervention to preemptive health maintenance, ultimately attenuating disease burden through upstream interception.

Despite the transformative potential of multimodal integration outlined above, its clinical translation faces substantial practical barriers. Interoperability between platforms remains largely undeveloped; data formats, acquisition protocols, and quality standards lack harmonization across modalities, creating integration bottlenecks that cannot be solved by algorithmic advances alone. Furthermore, regulatory pathways for integrated diagnostics remain undefined, and reimbursement frameworks for multimodal diagnostic algorithms do not yet exist. These represent structural prerequisites for clinical adoption rather than incremental engineering challenges, and their resolution will require coordinated efforts across technology developers, regulatory bodies, and healthcare systems. Addressing these prerequisites is essential if the promising technological advances reviewed herein are to achieve meaningful clinical impact.

## Data Availability

Not applicable.

## Abbreviations

The following abbreviations are used in this manuscript:

AD	Alzheimer’s Disease
PD	Parkinson’s Disease
ALS	Amyotrophic lateral sclerosis
HD	Huntington’s disease
Aβ	Amyloid beta
CSF	Cerebrospinal fluid
EEG	Electroencephalogram
PET	Positron emission tomography
MRI	Magnetic resonance imaging
NFTs	Neurofibrillary tangles
p-tau	Phosphorylated tau
